# Identification of N^7^-methylguanosine-related IncRNA signature as a potential predictive biomarker for colon adenocarcinoma

**DOI:** 10.3389/fgene.2022.946845

**Published:** 2022-08-29

**Authors:** Xiaomei Ma, Baoshun Yang, Yuan Yang, Guozhi Wu, Xiaoli Ma, Xiao Yu, Yingwen Li, Yuping Wang, Qinghong Guo

**Affiliations:** ^1^ The First Clinical Medical College, Lanzhou University, Lanzhou, China; ^2^ Department of Gastroenterology, The First Hospital of Lanzhou University, Lanzhou, China; ^3^ Key Laboratory for Gastrointestinal Diseases of Gansu Province, The First Hospital of Lanzhou University, Lanzhou, China; ^4^ General Surgery Ward 5, First Hospital of Lanzhou University, Lanzhou, China

**Keywords:** N^7^-methylguanosine, long non-coding RNAs, colon adenocarcinoma, prognosis, tumor immune microenvironment

## Abstract

N^7^-Methylguanosine (m7G) is an RNA modification serving as a key part of colon cancer development. Thus, a comprehensive analysis was executed to explore prognostic roles and associations with the immune status of the m7G-related lncRNA (m7G-RNAs) in colon adenocarcinoma (COAD). Identification of m7G-RNAs was achieved *via* Pearson’s correlation analysis of lncRNAs in the TCGA-COAD dataset and m7G regulators. A prognostic signature was developed *via* LASSO analyses. ESTIMATE, CIBERSORT, and ssGSEA algorithms were utilized to assess immune infiltration between different risk groups. Survival analysis suggested the high-risk group possesses poor outcomes compared with the low-risk group. According to the ROC curves, the m7G-RNAs signature exhibited a reliable capability of prediction (AUCs at 1, 3, and 5 years were 0.770, 0.766, and 0.849, respectively). Multivariate hazard analysis proved that the signature was an independent predictive indicator for OS. Moreover, the risk score was related to infiltration levels of naïve B cells, CD4^+^ memory T cells, and resting NK cells. The result revealed the prognostic value of m7G modification in COAD and provided a novel perspective on personalized immunotherapy strategies.

## Introduction

Colorectal cancer is among the top three tumors worldwide (Sung et al., 2021). Colon cancer has a higher incidence compared with rectal cancer (ratio = 2:1), and the ratio of the colon to rectal cases is ≥ 2 in developed countries and generally similar in developing countries (Labianca et al., 2010). Adenocarcinoma originating from epithelial cells of the colon mucosa is the most widely observed colon cancer subtype (Fleming et al., 2012). Despite advances in treatment modalities, colon cancer is ranked fourth in a list of cancer-related mortality causes in 2020 (5.8% of all sites) (Sung et al., 2021). It is urgent to elucidate molecular mechanisms and identify a novel molecular target for personalized management of colon adenocarcinoma (COAD).

As a post-translational modification that can be reversed, RNA methylation influences multiple biological processes, including splicing, nucleation, stability, and immunogenicity of RNA, in an epigenetic way. Meanwhile, the dysregulation of RNA methylation is necessary for human cancer development, especially gastrointestinal cancers (Xie et al., 2020; Zhang et al., 2021). There are several identified types of RNA methylation, including N^7^-methylguanosine (m7G), N6-methyladenosine (m6A), ribose methylations (Nm), N^1^-methyladenosine (m1A), and 5-methylcytosine (m5C) (Wiener and Schwartz, 2021). Among them, m7G is the modification of the seventh N of RNA guanine with a methyl group (Liu and Jia, 2014; Zhang et al., 2021).

Long non-coding RNAs (lncRNAs) play a significant role in pre-mRNA processing, gene transcription control, mature mRNAs’ transportation to corresponding cellular compartments, protein translation and turnover, and mRNA stability regulation (Riva et al., 2016). It has been reported that lncRNAs could mechanistically interact with the epigenetic machinery and facilitate tumorigenic chromatin remodeling to promote or suppress cancer progression (Begolli et al., 2019). Because of genome-wide expression patterns and tissue-specific expression characteristics, lncRNAs have potential application prospects in diagnostic biomarkers and therapeutic targets (Bhan et al., 2017).

Recently, more and more studies have found the interaction between lncRNA and RNA methylation in multiple cancer. For example, Zhang et al. found that ALKBH5 promoted GC invasion and metastasis through the demethylation of lncRNA NEAT1 (Sung et al., 2021). In colon cancer, METL14 downregulates the expression of lncRNA XIST by regulating the m6A level of XIST, thereby inhibiting the proliferation and metastasis of cancer cells (Labianca et al., 2010). Zhang et al. found that m5c modified H19 lncRNA may promote the occurrence and development of hepatocellular carcinoma by recruiting G3BP1 oncoprotein (Fleming et al., 2012). However, studies on the interaction between m7G and lncRNA are relatively scarce.

This study explored the predicting role of lncRNAs that are associated with m7G in the overall survival (OS) of COAD. A prognostic signature was developed based on 14 m7G-related lncRNAs (m7G-RNAs) in the development set, whereas its predictive value in the complete set and validation set was validated, respectively. The results revealed that the signature served as an independent survival predictor of COAD and the prediction accuracy was higher than that of clinical baseline features. Besides, the risk groups identified by the signature showed a significant difference in the immune microenvironment.

## Materials and methods

### Data collection and correlation analysis

**FIGURE 1:**
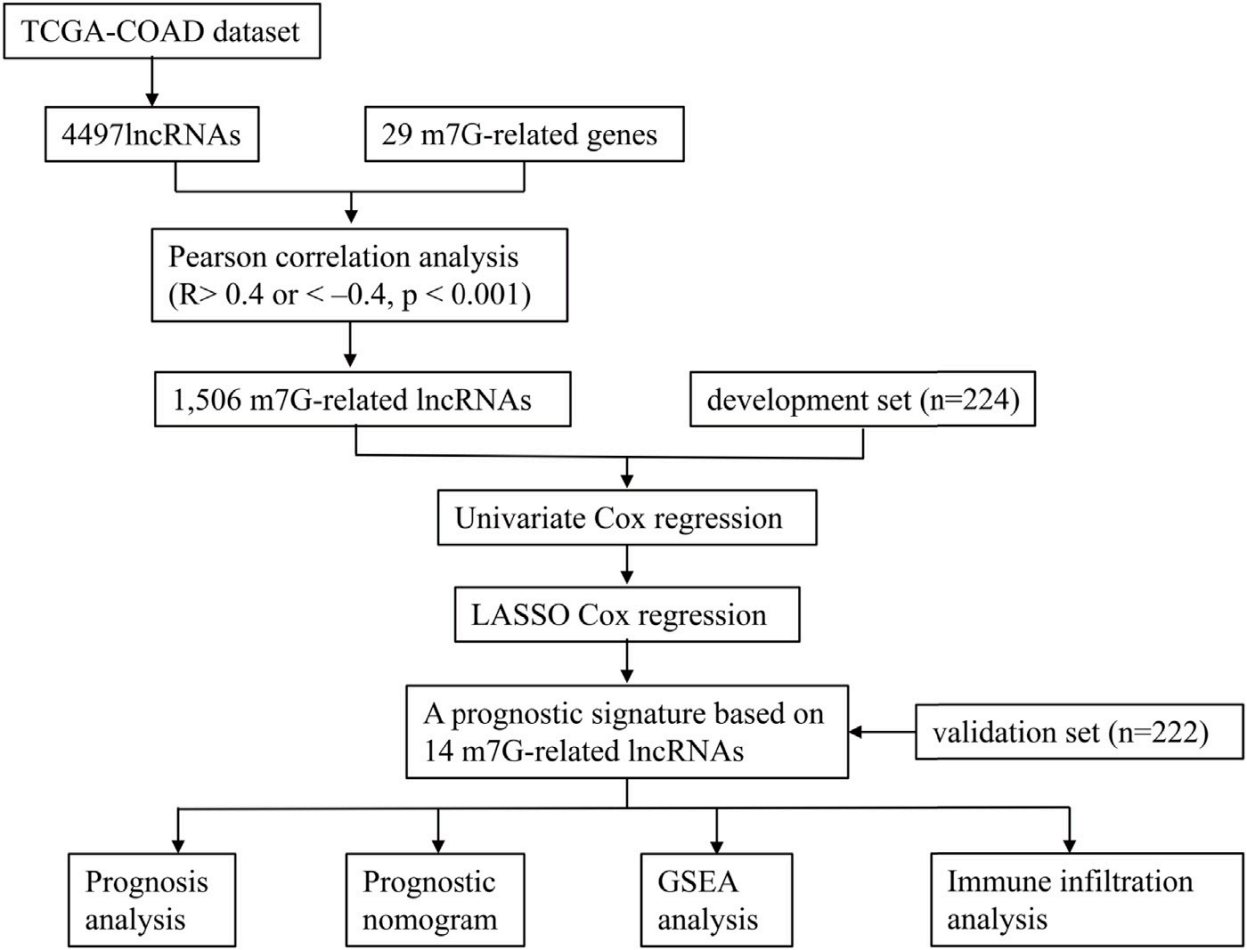
The flowchart of this study.

Figure 1 illustrates the analysis process of this study. The expression profile of the TCGA-COAD dataset was grouped into lncRNAs and protein-coding genes referring to human genome annotation data. m7G regulators were obtained from previous studies (Kiriakidou et al., 2007; Ng et al., 2015; Tomikawa, 2018) and MSigDB database (https://www.gsea-msigdb.org/gsea/msigdb). Three relevant gene sets were searched in the MSigDB database with “7-Methylguanosine” keywords, including “GOMF_M7G_5_PPPN DIPHOSPHATASE_ACTIVITY”, “GOMF_RNA _7_METHYLGUANOSINE_CAP BINDING”, and “GOMF RNA_CAP BINDING”. Finally, 29 m7G RNA methylation regulators (METTL1, DR4, NSUN2, DCP2, DCPS, NUDT10, NUDT11, NUDT16, NUDT3, NUDT4, NUDT4B, AGO2, CYFIP1, EIF4E, EIF4E1B, EIF4E2, EIF4E3, GEMIN5, LARP1, NCBP1, NCBP2, NCBP3, EIF3D, EIF4A1, EIF4G3, IFIT5, LSM1, NCBP2L, and SNUPN) were obtained, and their expression profiles were extracted from the TCGA-COAD datasets. Then, Pearson’s correlation analysis (PCA) was utilized to clarify the correlation between m7G-regulators and lncRNAs. The m7G-RNAs had an absolute value of correlation coefficients above 0.4 and a *p*-value less than 0.001.

### Establishment and validation of the m7G-RNAs signature

Samples were randomly divided into development and testing sets. The m7G-RNAs signature was developed and validated using the training and validation sets, respectively. The univariate hazard analysis was performed in the development set to identify the m7G-RNAs associated with OS. Then, prognostic lncRNAs were enrolled into the LASSO analysis to construct the m7G-RNAs signature. The risk score was determined as follows:

$$Risk\;score=\sum_{i}^{n}coef\left(i\right)*lncRNA\;expression\;\left(i\right).$$

The risk score of the validation set was determined using the same equation derived from the development set. Subsequently, the COAD samples were grouped into high-risk (HR) and low-risk (LR) groups, referring to the median of the risk score. The OS and PFS of the two groups were investigated based on the Kaplan–Meier (KM) survival curve. ROC curves and their areas under curve (AUC) were employed to evaluate the predictive accuracy of signature.

### Construction and identification of the predictive nomogram

Univariate and multivariate hazard analyses were used to confirm independent prognostic indicators. Then, a nomogram was developed based on clinical baseline features and m7G-RNAs signature with the “rms” R package. A calibration plot was employed to assess the agreement of predicted and actual survivals. The clinical efficacy of m7G-RNAs signature and nomogram was assessed by Decision Curve Analysis (DCA). Besides, the C-index was determined to assess the accuracy of predicted survival of the nomogram, m7G-RNAs signature, and clinical baseline features.

### Functional enrichment analyses

In order to clarify the potential molecular functions of m7G-RNAs signature, the Gene Set Enrichment Analysis (GSEA) and Gene Set Variation Analysis (GSVA) of the HR and LR groups were executed to evaluate differentially regulated GO or KEGG items between risk subgroups. FDR < 0.05 was considered significant. The “c5.go.v7.4.symbols.gmt” and “c2.cp.kegg.v7.4.symbols.gmt” genesets were used for reference.

### Tumor microenvironment analysis

In order to explore the role of the m7G-RNAs signature in the TME of COAD, the ESTIMATE algorithm was used to evaluate stromal and immune scores and the tumor purity of the two risk subgroups. The CIBERSORT algorithm was employed to assess the proportions of immune cell subtypes and the correlation of risk scores with immune cells. Then, the ssGSEA analysis was executed to compare differences in immune function and infiltration of immune cells of the HR and LR groups. Besides, the 18 genes that are related to the immune checkpoint (PDCD1, TIGIT, CD28, CD274, CD160, PDCD1LG2, CD244, BTN2A2, TMIGD2, LAG3, CD96, CD200, TNFRSF18, CD86, CD40, NRP1, CEACAM1, ADORA2A, CD44, CD70, HHLA2) were identified based on previous studies to explore their correlation with the m7G-RNAs signature (Fang et al., 2020; Kitsou et al., 2020; Li et al., 2021).

### Statistical analysis

The statistical analysis was processed in R software. The Perl programming language was used for data processing. Unless otherwise noted, *p* < 0.05 was considered statistically significant. In the figures, “*” represents *p* < 0.05, “**” represents *p* < 0.01, and “***” represents *p* < 0.001.

## Results

### m7G-RNAs acquisition in COAD

**FIGURE 2:**
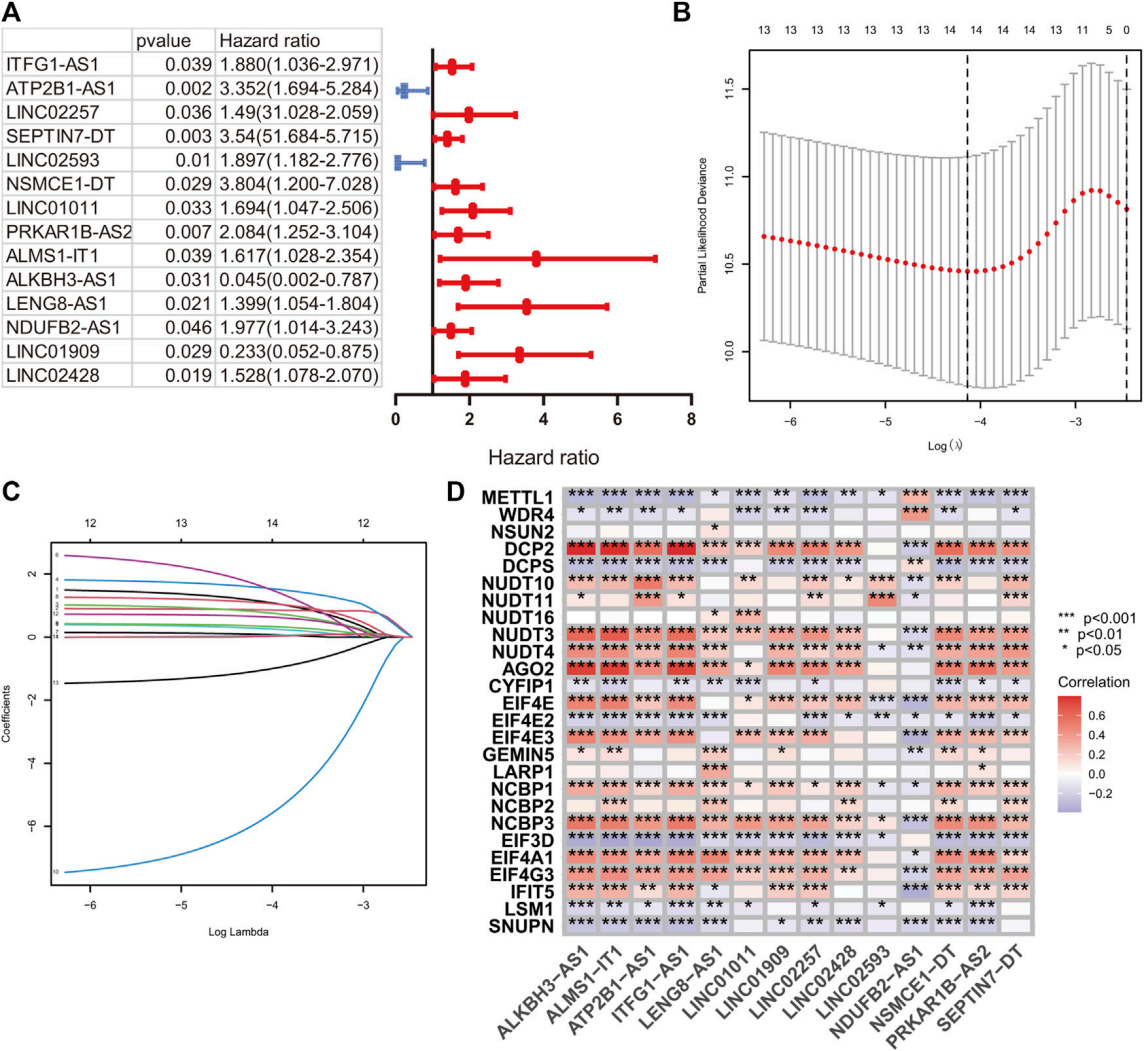
m7G-related signature construction. **(A)** The univariate hazard analysis of prognostic m7G-related lncRNAs in the development set. **(B and C)** LASSO analysis on 14 prognostic m7G-related lncRNAs in the development set. **(D)** The Pearson correlation analysis of m7G-related regulators and 14 prognostic m7G-related lncRNAs.

A total of 224 samples were assigned to the development set and 222 samples into the validation set. The clinical baseline features of the HR and LR groups are illustrated in Table 1. According to the lncRNA annotation data, 4,497 lncRNAs were identified in TCGA-COAD datasets. After evaluating the association between lncRNA and 29 m7G regulators, 1,506 m7G-RNAs were confirmed (|Pearson R|> 0.4 and *p* < 0.001). After that, a univariate hazard analysis was executed to explore the prognostic value of these lncRNAs. The hazard ratio values were all processed by log_2_ (value +1) to narrow down the absolute value range of the data. The result revealed that ITFG1-AS1, ATP2B1-AS1, LINC02257, SEPTIN7-DT, LINC02593, NSMCE1-DT, LINC01011, PRKAR1B-AS2, ALMS1-IT1, LENG8-AS1, NDUFB2-AS1, and LINC02428 were risky factors with hazard ratio greater than 1, whereas LINC01909 and ALKBH3-AS1 were protective factors with hazard ratio less than 1 (Figure 2A).

**TABLE 1:** Clinical baseline features of samples in training and validation sets.

Covariates	Type	Complete set	Development set	Validation set
Age	≤65	183 (41.03%)	95 (42.41%)	88 (39.64%)
	>65	263 (58.97%)	129 (57.59%)	134 (60.36%)
Gender	Female	212 (47.53%)	110 (49.11%)	102 (45.95%)
	Male	234 (52.47%)	114 (50.89%)	120 (54.05%)
Stage	Stage I-II	250 (56.05%)	116 (51.79%)	134 (60.36%)
	Stage III-IV	185 (41.48%)	103 (45.98%)	82 (36.94%)
	Unknown	11 (2.47%)	5 (2.23%)	6 (2.7%)

### Construction of prognostic signature

A LASSO analysis was conducted to generate the prognostic signature consisting of 14 identified m7G-related prognostic lncRNAs. Finally, all 14 lncRNAs were selected based on λ.min values, and the risk coefficient was calculated (Figures 2B,C). The risk coefficient of lncRNAs that comprise the prognostic signature is listed in Table 2. The risk score of each sample was calculated based on the risk coefficient and expression level of 14 m7G-related prognostic lncRNAs. Figure 2D demonstrated the correlation between the prognostic lncRNAs with m7G regulators. Samples with COAD were divided into HR or LR subgroups with the median cutoff of risk score.

**TABLE 2:** The risk coefficient of lncRNAs that comprise the prognostic signature.

lncRNA	Coefficient
ITFG1-AS1	1.141371803
ATP2B1-AS1	0.865146021
LINC02257	0.795554824
SEPTIN7-DT	1.573247835
LINC02593	0.276013057
NSMCE1-DT	1.632161275
LINC01011	0.08737383
PRKAR1B-AS2	1.092034967
ALMS1-IT1	0.349485749
ALKBH3-AS1	−5.84063052
LENG8-AS1	0.049777684
NDUFB2-AS1	0.650094369
LINC01909	−1.06225964
LINC02428	0.019695671

### Evaluation of the prognostic signature

**FIGURE 3:**
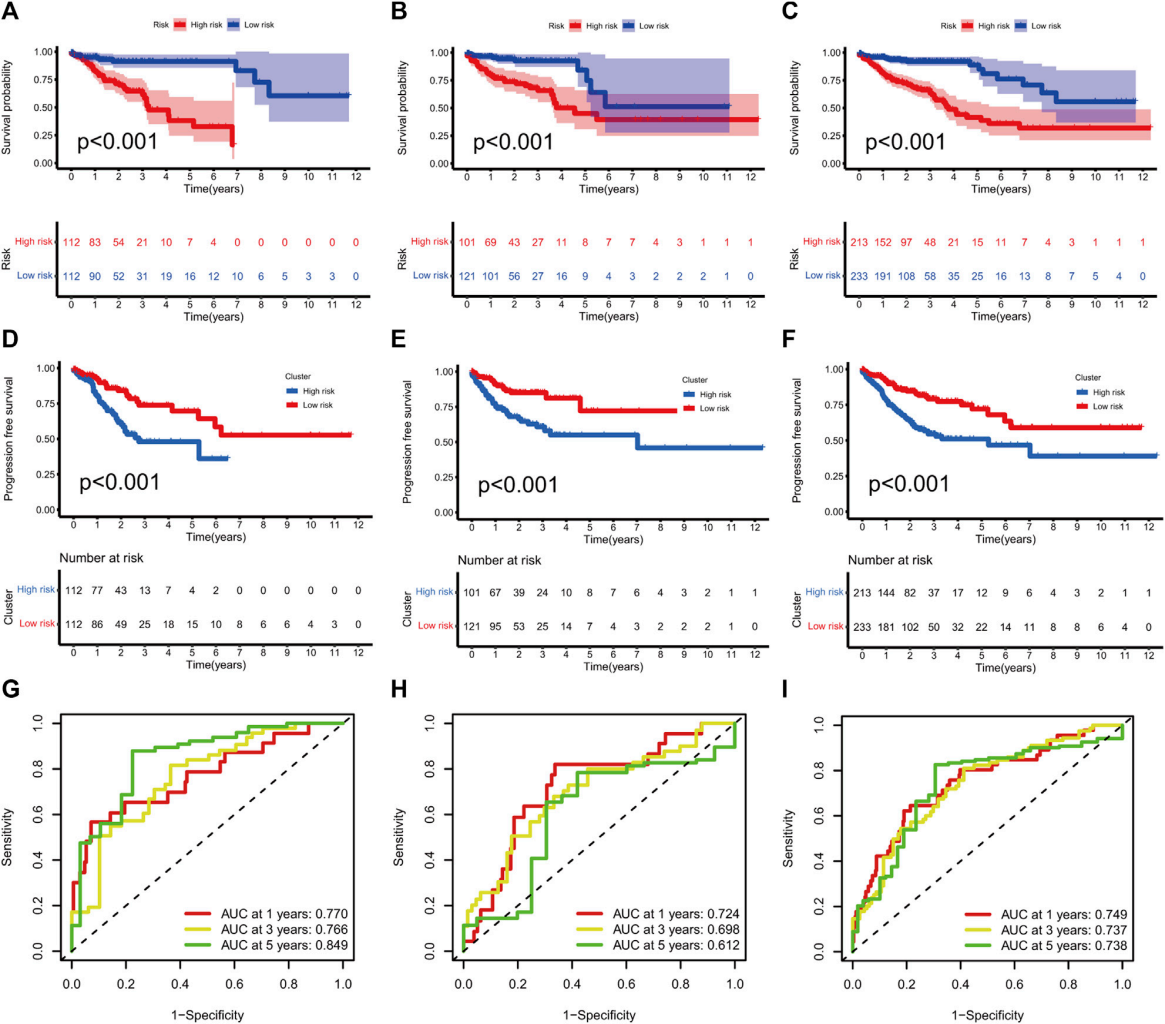
m7G-related signature validation. **(A–C)** Overall survival analysis between risk subgroups in development set (**A**, *p* < 0.001), validation set (**B**, *p* < 0.001), and complete set (**C**, *p* < 0.001). **(D–F)** Progression-free survival analysis between risk subgroups in development set (**D**, *p* < 0.001), validation set (**E**, *p* < 0.001) and complete set (**F**, *p* < 0.001). **(G–I)** ROC curves at 1, 3, and 5 years in the development set **(G)**, validation setm **(H)** and complete set **(I)**.

**FIGURE 4:**
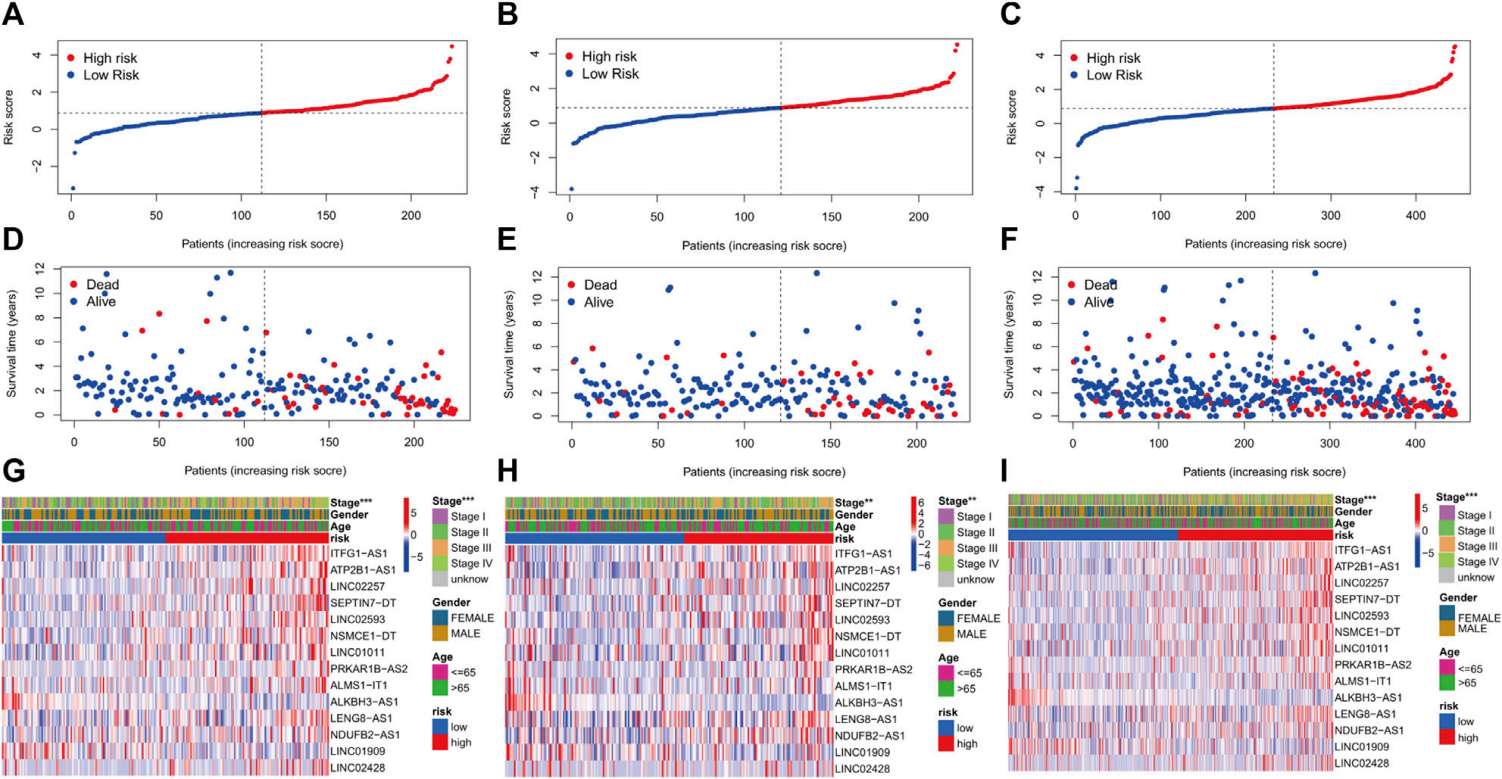
m7G-related prognostic signature in COAD. **(A–C)** Distribution of samples’ risk scores in risk subgroups in the development set **(A)**, validation set **(B),** and complete set **(C)**. **(D–F)** Survival status for samples in the development set **(D)**, validation set **(E),** and complete set **(F)**. **(G–I)** Distribution heatmap of prognostic m7G-related lncRNAs and clinical baseline features in risk subgroups in the development set (**G**, stage: *p* < 0.001), validation set (**H**, stage: *p* < 0.01), and complete set (**I**, stage: *p* < 0.001). (*p* < 0.001,“***”; *p* < 0.01,“**”; *p* < 0.05,“*”).

**FIGURE 5:**
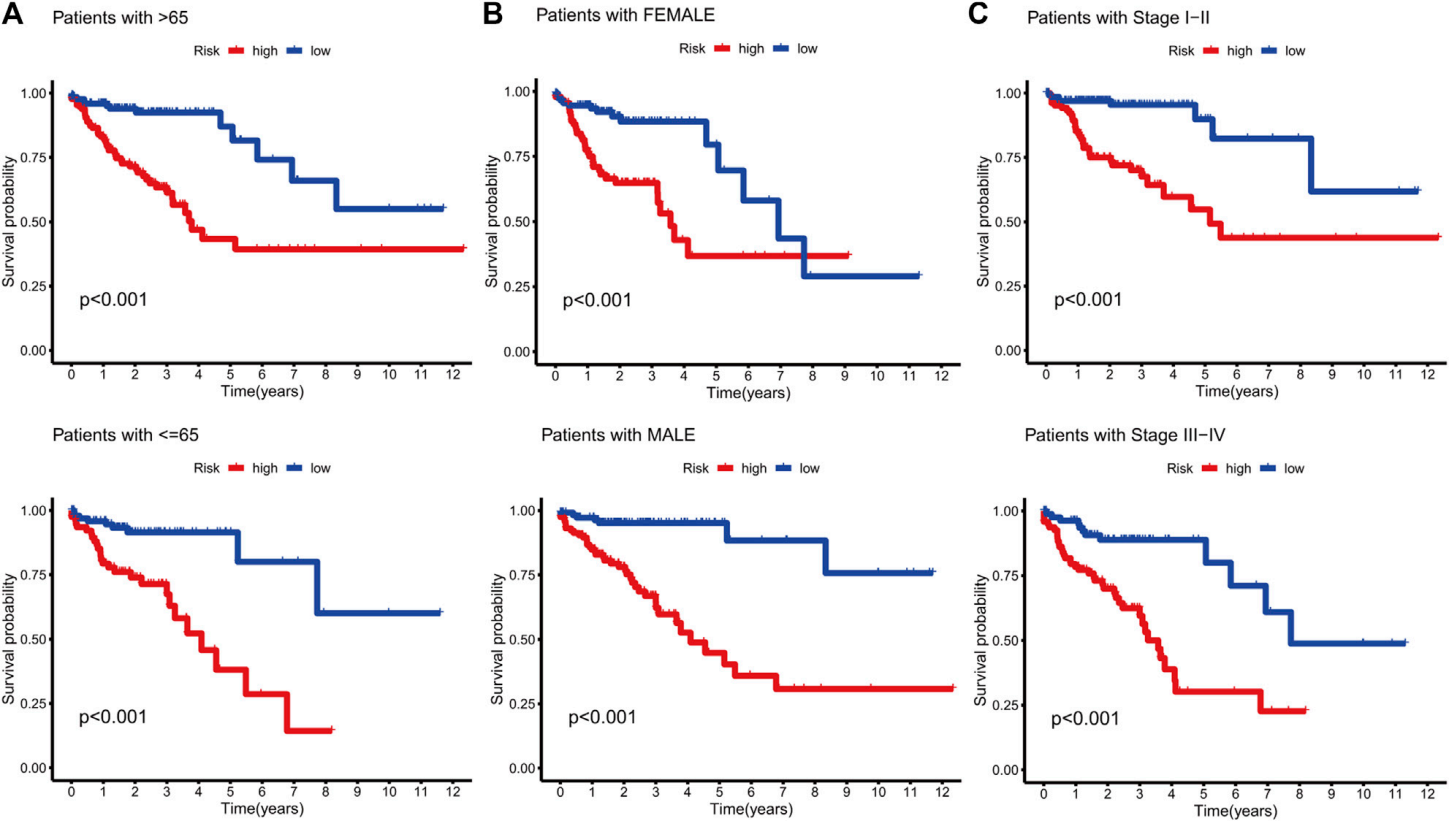
Survival analysis in clinical subtypes. **(A)** KM curve of age-differentiated clinical subtypes (>65 years and ≤65 years). **(B)** KM curve of gender-differentiated clinical subtypes (female and male). **(C)** KM curve of stage-differentiated clinical subtypes (Stages I-II and Stages III-IV).

KM curves demonstrated that the HR group had a poor OS and PFS than the LR group (Figures 3A–F). The ROC-AUC at 1, 3, and 5 years was 0.770, 0.766, and 0.849 in the development set; 0.724, 0.698, and 0.612 in the validation set; and 0.749, 0.737, and 0.739 in the complete set, respectively, indicating good prediction accuracy of m7G-RNAs signature in COAD survival (Figures 3G–I). According to the distribution plot, samples in the HR group had higher risk scores than those in the LR group (Figures 4A–C). The scatter plot showed a shorter OS of the HR group than the LR group (Figures 4D–F). Besides, the heatmap showed significant differences in tumor stage of risk subgroups (Figures 4G–I). The signature of m7G-RNAs also had prognostic significance in clinical subtypes stratified by age, gender, and tumor stage (Figure 5). These results suggest that the m7G-related prognostic model can effectively predict the OS of patients and is significantly correlated with tumor stage in COAD. In clinical practice, this model may effectively identify COAD patients with a high risk of death and greatly help individualized tumor treatment.

### Identification of prognostic nomogram

**FIGURE 6:**
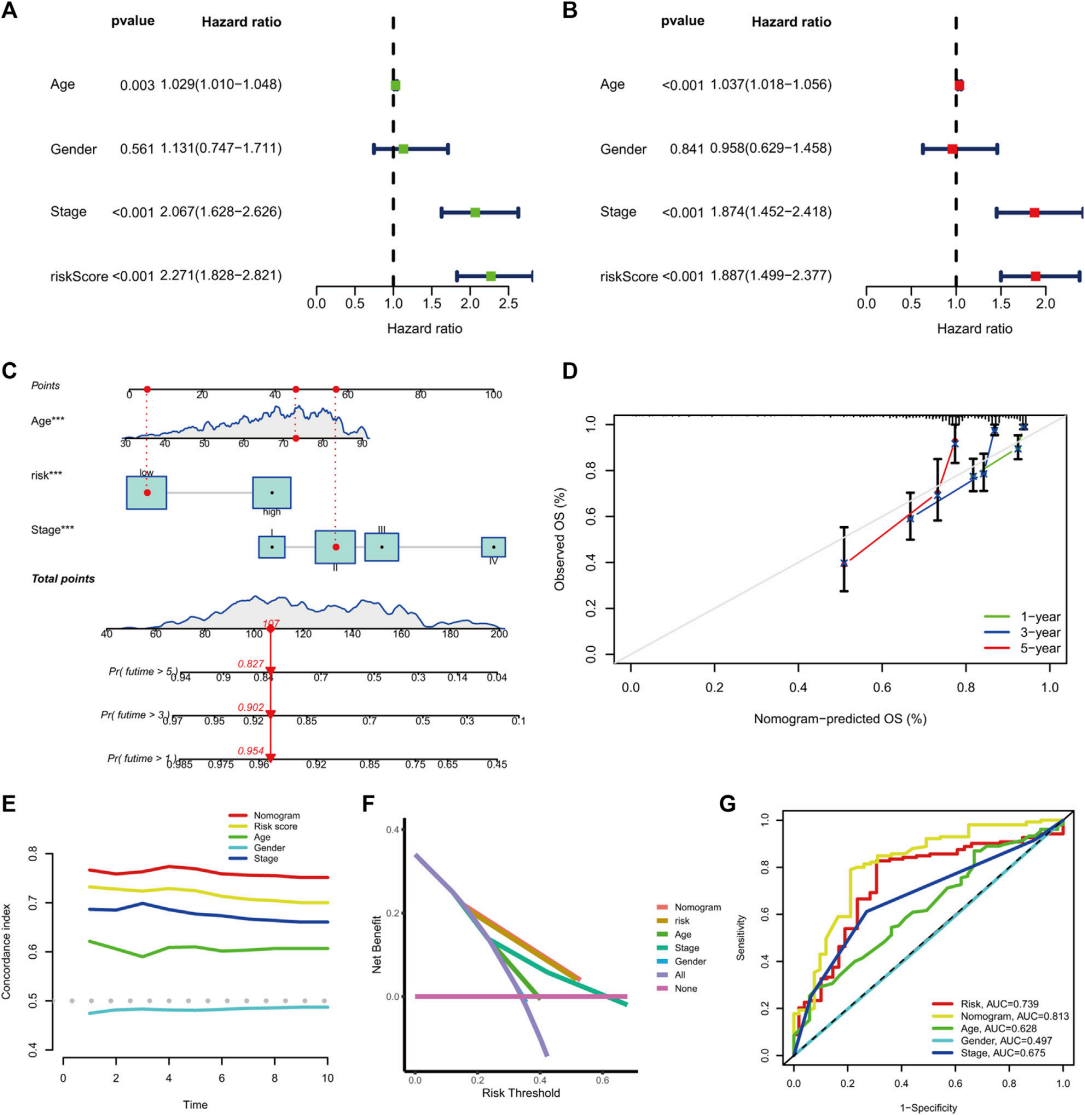
The prognostic nomogram generation and validation. **(A)** Univariate hazard analysis of the prognostic score and clinical baseline features. **(B)** The multivariate hazard analysis of the prognostic score and clinical baseline features. **(C)** Establishment of the prognostic nomogram. **(D)** The calibration curves of the nomogram signature at 1-, 3-, and 5-year OS. **(E)** C-index of the prognostic nomogram, prognostic signature, and clinical baseline features from 1 to 10 years. **(F)** DCA curves for prognostic nomogram, prognostic signature, and clinical baseline features. **(G)** ROC curve at 5 years for prognostic nomogram, prognostic signature, and clinical baseline features.

The univariate hazard analysis indicated that age (HR = 1.029, *p* = 0.003), stage (HR = 2.067, *p* < 0.001), and m7G-RNAs (HR = 2.271, *p* < 0.001) were risk factors for COAD (Figure 6A). Subsequently, the multivariate hazard analyses further confirmed that age (HR = 1.037, *p* < 0.001), stage (HR = 1.874, *p* < 0.001), and the prognostic signature (HR = 1.887, *p* < 0.001) were independent predictors in COAD (Figure 6B). Then, the independent predictors were incorporated to build the prognostic nomogram. Samples had a corresponding nomogram score according to the original risk score and clinical baseline features, including age and stage (Figure 6C). Calibration curves demonstrated that prognostic nomogram may lead to the high consistency of predicted and actual OS (Figure 6D). The C-index indicated that the prognostic nomogram and lncRNA signature had a high prediction accuracy (Figure 6E). The DCA revealed that the prognostic nomogram and the lncRNA signature had great potential for clinical prognosis application (Figure 6F). The ROC curves at 5 years showed that the prognostic nomogram (AUC = 0.813) and the lncRNA signature (0.739) had a more predictive ability of accuracy compared to the stage (AUC = 0.675) and age (AUC = 0.628) (Figure 6G).

### Functional annotation analysis

**FIGURE 7:**
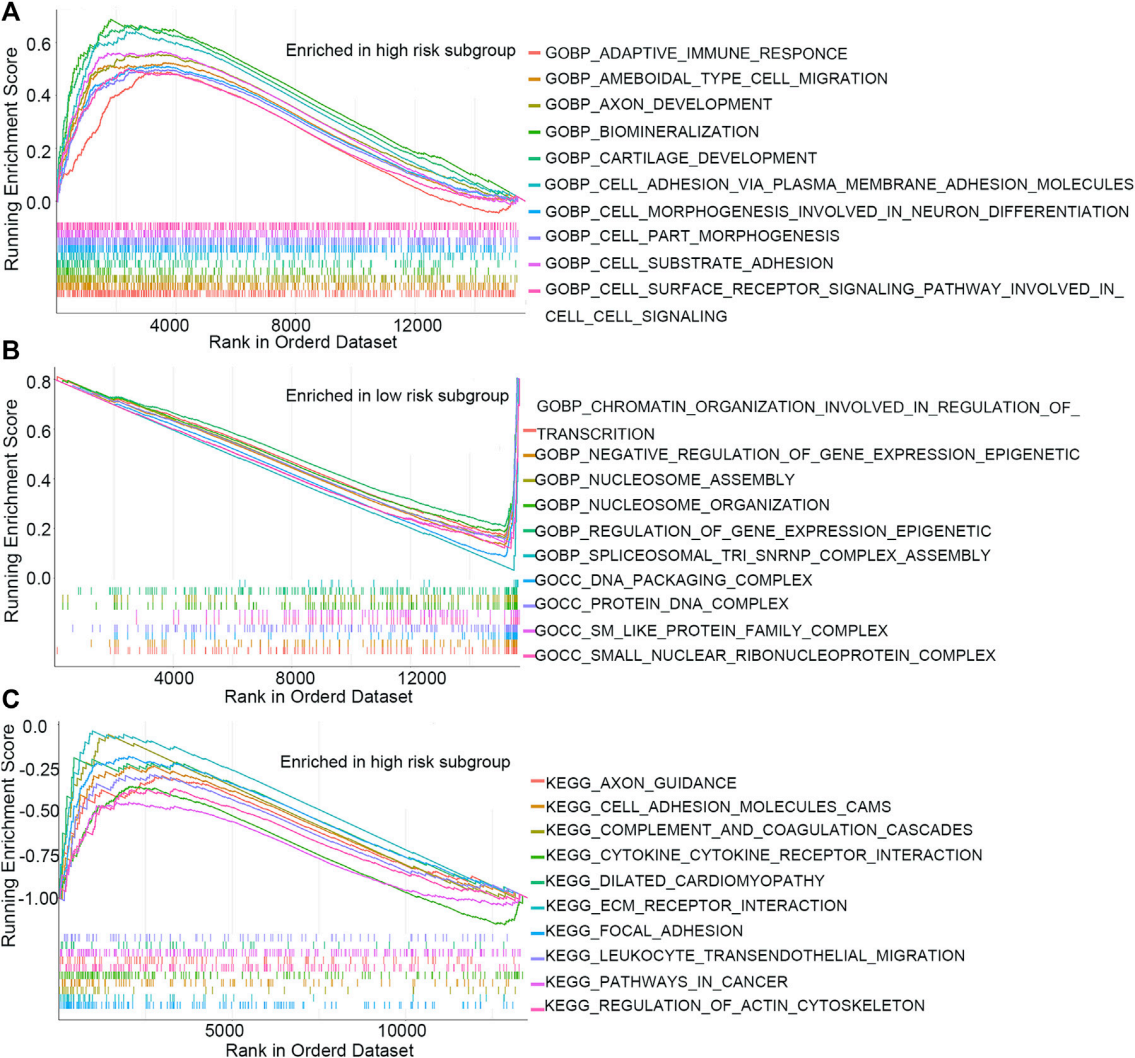
GSEA analysis. **(A)** Top 10 GO items in the high-risk subgroup. **(B)** Top 10 GO items in the low-risk subgroup. **(C)** Top 10 KEGG items in the high-risk subgroup.

GSEA and GSVA were performed to investigate the underlying biological process that the m7G-RNAs signature may be involved in COAD. The top pathways or functions of GSEA are shown in Figure 7. Several enrichment pathways that are significantly associated with cancer were noted, including peroxisome proliferator-activated (PPAR) signaling and cell adhesion pathways in the HR group and enrichment of DNA packaging- and nucleosome-related signaling pathways in the LR group. The results contribute to a thorough understanding of the regulatory mechanism of m7G-RNAs signature in COAD. The top 20 different KEGG and GO items identified with GSVA were respectively displayed with heatmaps (Supplementary Figure S1). In GSVA analysis, the p53 signaling pathway, cell cycle, glycolysis gluconeogenesis, and other cancer-related pathways were identified.

### Immune landscape of risk subgroups

**FIGURE 8:**
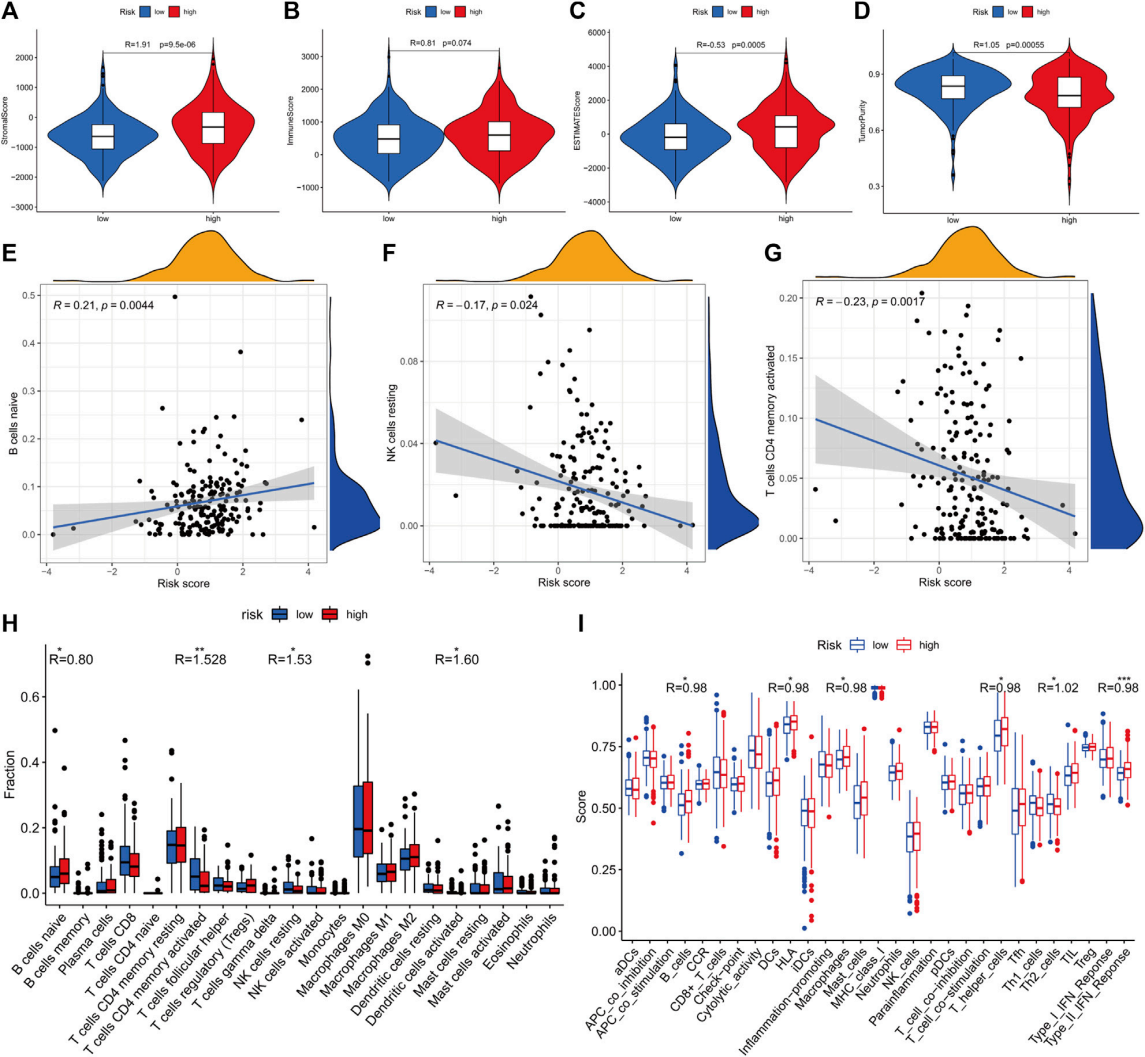
The landscape of tumor microenvironment. **(A–D)** Stromal, immune, and ESTIMATE scores and tumor purity in the two risk subgroups. **(E–G)** The correlation between the prognostic signature with naïve B cells, resting NK cells, and CD4 memory active T cells. **(H)** CIBERSORT analysis of immune cells. **(I)** ssGSEA scores of immune cells and activities (*p* < 0.001,“***”; *p* < 0.01,“**”; *p* < 0.05,“*”).

**FIGURE 9:**
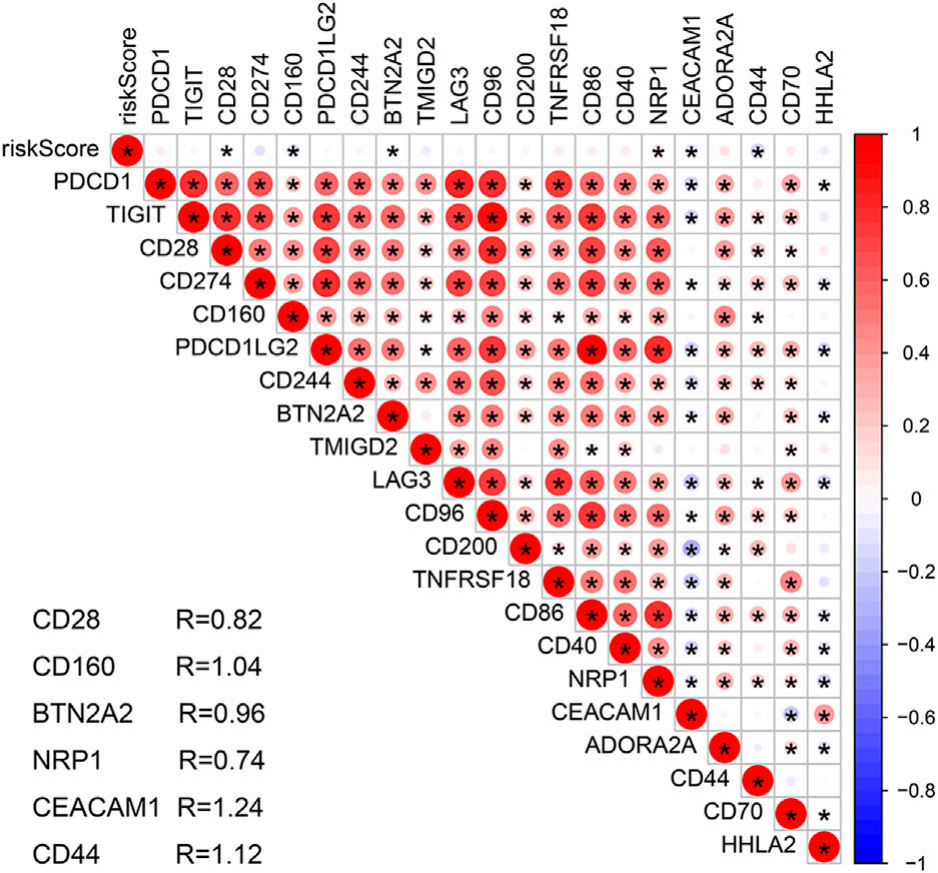
Correlation analysis of the prognostic signature and immune checkpoint-related genes.

The stromal and ESTIMATE scores of the HR subgroup were significantly higher than those of the LR group. In contrast, the tumor-purity score of the HR subgroup was lower than that of the LR group, suggesting that stromal cells play a key role in tumor progression of COAD (Figures 8A–D). CIBERSORT analysis demonstrated that m7G-RNAs signature positively correlated with the infiltration of naïve B cells and negatively correlated with CD4^+^ memory T cells (CD4TC) and resting NK cells (Figures 8E–G). Consistently, naïve B cells exhibited infiltration abundance in the HR group, whereas CD4TC and resting NK cells exhibited infiltration abundance in the HR group (Figure 8H). Besides, the ssGSEA analysis revealed that type II IFN response and the expression of B cells, human leukocyte antigen (HLA), macrophages, and helper T cells were superior in the HR group, whereas Th2 cells were inferior in the HR group (Figure 8I). With regard to the immune checkpoint, the signature of m7G-RNAs was positively related to CD28, BTN2A2, and NRP1 but negatively related to CD44, CD160, and CEACAM1 (Figure 9). The differences in the immune status of HR and LR groups indicated that the immune microenvironment served as a significant participant in the development of COAD.

## Discussion

As a positively charged post-transcriptional modification, m7G regulates most steps of mRNA’s life cycle, such as translation and splicing (Zhang et al., 2019). m7G is present in not only mRNA caps, but also tRNAs, rRNAs, and some internal positions within mRNAs (Pandolfini et al., 2019; Zhang et al., 2019). It has been found that m7G plays an indispensable role in gene expression and cell viability (Chen et al., 2019). RNA methylation is regulated by a crowd of RNA-modifying proteins. Aberrant of RNA modification and corresponding proteins have been identified in tumor tissues (Xie et al., 2020). RNA-modifying proteins related to cancer could regulate the metabolism of RNAs and the expressions of genes necessary for tumor proliferation, transformation, and invasion (Xie et al., 2020). In the present study, the prognostic value of m7G-RNAs and their effects on the immune microenvironment were thoroughly investigated in COAD.

First, m7G-RNAs were confirmed *via* the Pearson correlation analysis, resulting in the acquisition of 4,497 lncRNAs related to m7G. Then, a univariate hazard analysis was performed to determine m7G-RNAs with prognostic values. Among them, 14 RNAs were associated with OS outcomes of COAD. After that, LASSO analysis was conducted to develop an m7G-RNAs signature based on 14 m7G-RNAs. KM survival analysis indicated that the OS and PFS of the HR group were shorter than those of the LR group. Further ROC analysis results suggest that the prognostic signature has high accuracy in predicting OS of COAD. In addition, multivariate hazard analysis proved that m7G-RNAs signature, tumor stage, and age of samples were independent prognostic indicators for reliable prediction of OS in COAD. Besides, the OS of COAD was quantitatively predicted using a prognostic nomogram. Overall, the prognostic signature and nomogram identified in the present study showed satisfactory predictive accuracy for outcomes of the COAD sample better than clinical baseline features.

GSEA results demonstrated significant enrichment of the peroxisome proliferator-activated receptor (PPAR) signaling and cell adhesion pathways in the HR group and enrichment of DNA packaging- and nucleosome-related signaling pathways in the LR group. Studies have found that cell adhesion was associated with major characteristics of cancers, such as anchorage-independent growth, immune evasion, and metastatic dissemination, which were critical for cancer progression (Läubli and Borsig, 2019; Janiszewska et al., 2020). Variations in the cell-extracellular matrix (ECM) and inter-cell adhesions contribute to intravasation, invasion, extravasation, and anchorage-independent survival in the circulation of cancer cells, as well as their homing in a distant organ (Sousa et al., 2019). The PPAR receptors, which are members of the super-family of nuclear receptors, serve as ligand-inducible transcription factors in metabolisms of glucose and lipid (Mirza and AlthagafiShamshad, 2019). Moreover, the expression of PPARs is observed in immune cells and is of great importance in the differentiation of immune cells (Christofides et al., 2021). Several clinical trials have attempted to use PPARs as a therapeutic target for cancer (Wagner and Wagner, 2020). Nucleosomes serve as a fundamental structural unit of chromatin generated by DNA and histones in eukaryotic cells (Xu and Zhu, 2010). The chromosomal DNA was packaged into nucleosome strings, resulting in condensation and organization of the genome, which are essential for tight regulation of gene expressions by eukaryotic cells (Clapier and Cairns, 2009; Venkatesh and Workman, 2015). Nucleosomes protect the genome from DNA damaging agents and deposit a myriad of epigenetic signals (Cutter and Hayes, 2015). It has been reported that circulating nucleosomes are potential liquid biopsies that facilitate cancer detection at an early stage and treatment response monitoring (McAnena et al., 2017).

Studies have confirmed the regulatory effect of RNA methylation on the immune microenvironment in tumors (Xie et al., 2020). In the current study, the HR group identified by our prognostic signature had higher stromal cell proportions and lower tumor purity than the LR group. The risk score has a positive relationship with the infiltration levels of naïve B cells, whereas it is negatively related to CD4 memory active T cells and resting NK cells. Besides, the degrees of type II IFN response and human leukocyte antigen (HLA) were higher in the HR group. Tumor heterogeneity was previously thought to be related to abnormal genetic mutations, but nowadays, increasing studies indicate cancers also vary with the microenvironmental component, stromal cell infiltration, and activation states (Quail and Joyce, 2013). TME helps maintain tumor stemness and facilitates tumor malignant activates, such as angiogenesis, metastasis, and chronic inflammation (Denton et al., 2018). It has been demonstrated that cancer-associated fibroblasts (CAFs), which are the dominant reactive stroma type, had a pro-tumorigenic effect by secreting growth factors, cytokines, chemokines, and H_2_O_2_ and degrading ECM (Liao et al., 2019). Besides, genetic mutations in both type II IFN and its receptor could induce colorectal cancer development (Di Franco et al., 2017). Therefore, it is promising to explore the variability of immune profiles between tumor subtypes and identify potential prognostic or therapeutic targets.

Our study still had some limitations. Firstly, our prognostic signature was established based on the TCGA database and lacked a patient cohort to further verify its value. Secondly, our conclusion is only based on data analysis, and further experiments are needed to explore the mechanisms by which m7G-related lncRNAs influence the development of COAD.

## Conclusion

In conclusion, our study comprehensively analyzed the predictive value of m7G-associated lncRNAs in COAD prognosis. We developed a 14-lncRNA signature and a prognostic nomogram based on m7G-related lncRNA and clinical baseline features, both showing high predictive accuracy of survival time in COAD samples. Further analysis indicated a valid correlation between the prognostic signature and immune cell infiltration, immune pathways, and immune checkpoints.

## Data Availability

The datasets analyzed for this study can be found in The Cancer Genome Atlas (https://portal.gdc.cancer.gov) and MSigDB database (https://www.gsea-msigdb.org/gsea/msigdb).
