# Supplementary figures and images for: Identification of N7-methylguanosine-related IncRNA signature as a potential predictive biomarker for colon adenocarcinoma
Source: Front Genet. 2022 Aug 29;13:946845. doi: 10.3389/fgene.2022.946845 (PMC9465161; doi:10.3389/fgene.2022.946845)

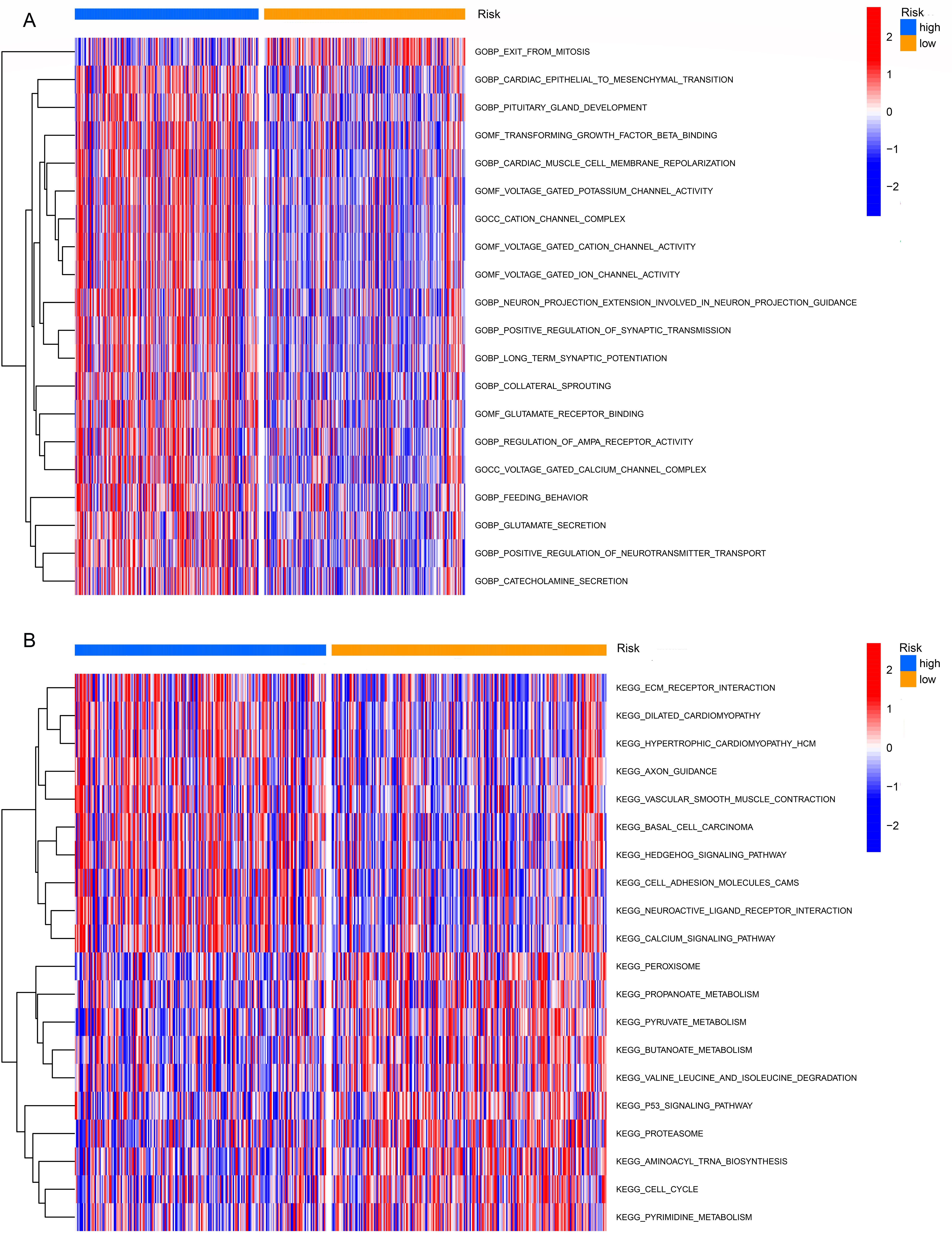

Supplement: Supplementary file 1 [file Image1.JPEG]
